# Redox Interactions of Vitamin C and Iron: Inhibition of the Pro-Oxidant Activity by Deferiprone

**DOI:** 10.3390/ijms21113967

**Published:** 2020-05-31

**Authors:** Viktor A. Timoshnikov, Tatyana V. Kobzeva, Nikolay E. Polyakov, George J. Kontoghiorghes

**Affiliations:** 1 Institute of Chemical Kinetics & Combustion, 630090 Novosibirsk, Russia; kobzeva@mail.ru (T.V.K.); polyakov@kinetics.nsc.ru (N.E.P.); 2 Postgraduate Research Institute of Science, Technology, Environment and Medicine, CY-3021 Limassol, Cyprus

**Keywords:** vitamin C, ascorbic acid, iron toxicity, iron chelation, deferiprone, Fenton reaction, hydroxyl radical, ascorbyl radical, EPR spin trapping, TMIO spin trap

## Abstract

Ascorbic acid (AscH_2_) is one of the most important vitamins found in the human diet, with many biological functions including antioxidant, chelating, and coenzyme activities. Ascorbic acid is also widely used in medical practice especially for increasing iron absorption and as an adjuvant therapeutic in iron chelation therapy, but its mode of action and implications in iron metabolism and toxicity are not yet clear. In this study, we used UV–Vis spectrophotometry, NMR spectroscopy, and EPR spin trapping spectroscopy to investigate the antioxidant/pro-oxidant effects of ascorbic acid in reactions involving iron and the iron chelator deferiprone (L1). The experiments were carried out in a weak acidic (pH from 3 to 5) and neutral (pH 7.4) medium. Ascorbic acid exhibits predominantly pro-oxidant activity by reducing Fe^3+^ to Fe^2+^, followed by the formation of dehydroascorbic acid. As a result, ascorbic acid accelerates the redox cycle Fe^3+^ ↔ Fe^2+^ in the Fenton reaction, which leads to a significant increase in the yield of toxic hydroxyl radicals. The analysis of the experimental data suggests that despite a much lower stability constant of the iron–ascorbate complex compared to the FeL1_3_ complex, ascorbic acid at high concentrations is able to substitute L1 in the FeL1_3_ chelate complex resulting in the formation of mixed L1_2_AscFe complex. This mixed chelate complex is redox stable at neutral pH = 7.4, but decomposes at pH = 4–5 during several minutes at sub-millimolar concentrations of ascorbic acid. The proposed mechanisms play a significant role in understanding the mechanism of action, pharmacological, therapeutic, and toxic effects of the interaction of ascorbic acid, iron, and L1.

## 1. Introduction

**Scheme 1:**
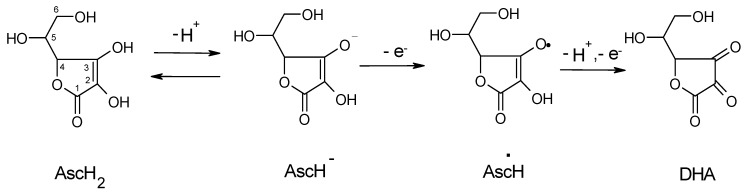
The structure of ascorbic acid and the main products of redox reactions. Ascorbic acid—AscH_2._; ascorbate anion—AscH^−^; ascorbyl radical—AscH^•^; dehydroascorbic acid—DHA.

Vitamin C or ascorbic acid (AscH_2_, Scheme 1) is a powerful water-soluble antioxidant, found in nature and present in fresh fruit and vegetables [1,2,3]. Ascorbic acid is also known as a reducing agent and a radical scavenger. As a radical scavenger AscH_2_ provides effective protection of membranes and proteins against oxidation by reactive oxygen species (ROS): superoxide (O_2_^•−^), hydrogen peroxide (H_2_O_2_), hydroxyl radicals (•OH), peroxyl radicals (•OOH), and singlet oxygen ^1^O_2_, especially at high concentrations [4,5]. 

However, ascorbic acid is a multifaceted compound that can act as a chelator and also as a source of free radicals [6,7,8,9,10]. The role of ascorbic acid as a chelator in iron and copper overload conditions is widely discussed [11,12,13]. In this regard, the literature intensively discusses the possibility of using ascorbic acid to treat advanced cancer in combination with anticancer agents [14,15,16,17,18,19,20,21,22,23,24,25]. In particular, some researchers have demonstrated biological validity and are ready to study the potential value of ascorbic acid in the treatment of cancer in humans [17]. However, the evidence that ascorbic acid could help human cancer patients is still thin and requires additional study. The results of published studies fail to prove that there is a clinically relevant positive effect of ascorbic acid supplementation in cancer patients in general on the overall survival, clinical status, quality of life and performance status [1,14,26].

Iron is an essential element of life. It plays an important role in many cellular processes including energy production, oxygen transport, DNA synthesis, as well as in many other important metabolic processes involving iron-containing enzymes [6,10]. The catalytic activity of iron stems from its redox properties, namely the ability to alternate between two oxidation states, Fe^3+^ and Fe^2+^, acting as an electron donor or acceptor. On the other hand, an excess of iron is toxic because it can cause the formation of an excessive amount of ROS, which can subsequently cause biomolecular, subcellular, cellular, and tissue damage. Excess toxic iron has been identified in many diseases, such as age-related macular degeneration, age-related Alzheimer’s and Parkinson’s diseases, as well as cancer [6,26,27,28,29,30,31,32]. 

**Figure 1:**
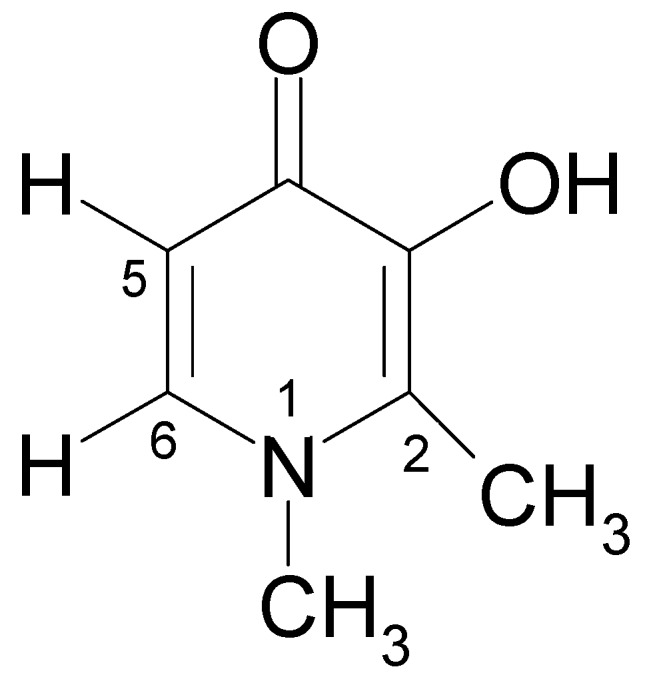
The chemical structure of deferiprone (L1).

Iron chelation therapy is widely used to prevent poisoning by removing excess iron caused from repeated blood transfusions and other iron overload diseases. It includes the use of various chelating agents that bind and remove excess iron from the body. [6,26,32,33,34]. In this regard, numerous studies have been conducted to elucidate the redox activity of iron in chelate complexes [35,36,37,38,39]. As an example, we recently demonstrated the inhibition of iron- and copper-induced hydroxyl radical production by the water-soluble chelator deferiprone (L1, Figure 1) [39,40]. L1 is an effective iron-chelating drug developed for the treatment of iron overload toxicity in thalassaemia and other iron-related toxicity conditions. L1 is orally active and widespread in the body, thus binding toxic iron in many tissues and organs, including the brain [41,42,43]. Moreover, L1 is supposed to be an effective antioxidant that prevents oxidative stress and biomolecular, subcellular, cellular, and tissue damage caused mainly by iron and copper-induced free radical formation [43]. The physicochemical and coordination properties of the L1 complex with iron have been previously studied in detail by many investigators [41,42,44,45,46].

In contrast, ascorbic acid is generally regarded as a weak chelating agent and cannot compete in iron binding with L1 and other stronger chelators. The coordination chemistry of ascorbic acid has been extensively studied in response to problems associated with its hydrolysis and redox reactions, as well as with the instability of its complexes [47,48,49,50,51,52,53]. As a weak dibasic acid (pKa1 = 4.25 and pKa2 = 11.79), at physiological pH ascorbic acid exists as monoanion (AscH^−^) with deprotonation of O(3)–H (see Scheme 1). The monoanionic form is quite stable due to the delocalisation of the negative charge between the oxygen atoms O(1) and O(3). Although ascorbic acid has several donor atoms capable of forming an iron complex (see Scheme 1), the interaction of ascorbic acid with ions of iron or other metals is mainly thought to occur by chelation through O(3) and O(2) nuclei accompanied by hydrogen displacement [53].

Some researchers have used time-resolved stop-flow spectrophotometry to follow the reactions of ascorbic acid with Fe^3+^ in aqueous solutions and found evidence of blue intermediates in the reduction pathway of iron at low pH [47]. It has been shown that the reaction proceeds on a millisecond time scale with the subsequent formation of the oxidation product of ascorbic acid, namely dehydroascorbic acid (DHA) (see Scheme 1). These and other researchers pointed to the rapid reduction of Fe^3+^ via intramolecular electron transfer with simultaneous production of the ascorbyl radical (AscH^●^) [38,39,40,47].

It is assumed that some of the above mechanisms occur in plants, where iron is delivered to embryos in the form of ferric complexes with citrate and malate [48]. In this context, embryos efflux a large amount of ascorbic acid which chemically reduces and releases Fe^3+^ from citrate and malate complexes. It was concluded that ascorbic acid plays a key role in the chemical reduction and transport of Fe^2+^. The interaction of ascorbic acid with iron complexes of (1,3)-bis-(2-hydroxybenzamido)propane and N,N’-ethylene-bis-salicylamide has also been studied [45,46]. It was shown that these complexes undergo a quick reaction with ascorbic acid to form ternary complexes in which AscH^−^ is chelated with the Fe^3+^ centre. Furthermore, it was also reported that these ternary complexes undergo intramolecular electron transfer with the formation of Fe^2+^ and dehydroascorbic acid [51]. Other investigators have applied density functional theory calculations to elucidate the structures and relative stability of Fe^3+^ complexes with various ligands (aminoguanidine, pyridoxamine, EDTA, and ascorbic acid). The distorted neutral octahedral complex containing one iron atom and three molecules of ascorbic acid was found to be the most stable, but it was still energetically less stable by 92.7 kcal/mol than the EDTA complex. However, it is worth noting that in an in vivo study the acute pro-oxidant effects of ascorbic acid in EDTA chelation therapy were demonstrated when patients were administered a standard EDTA cocktail solution with 5 g of sodium ascorbate [52].

Ascorbic acid is sold as a dietary supplement and is used daily by millions of people worldwide. It is also widely used in medical practice especially for increasing iron absorption and as an adjuvant in iron chelation therapy. Nevertheless, the mode of action of ascorbic acid and its interactions with iron, other metals, and natural or synthetic chelators including chelating drugs is not yet fully clarified [5].

In the present study, we applied a set of physicochemical methods (UV–Vis spectrophotometry, nuclear magnetic resonance (NMR) and electron paramagnetic resonance (EPR) spin trapping techniques) to answer major questions which may have wide implications in the dietary and clinical use of ascorbic acid in conjunction with iron. One of these questions is whether ascorbic acid works as a pro-oxidant in the biologically important Fenton reaction. Another question is whether ascorbic acid is able to compete in iron-binding and antioxidant activity with the iron-chelating drug L1 at low and high concentrations.

## 2. Results

### 2.1. UV–Vis Optical Study of the Interaction of Ascorbic Acid with the Iron-Deferiprone Chelate Complex

**Figure 2:**
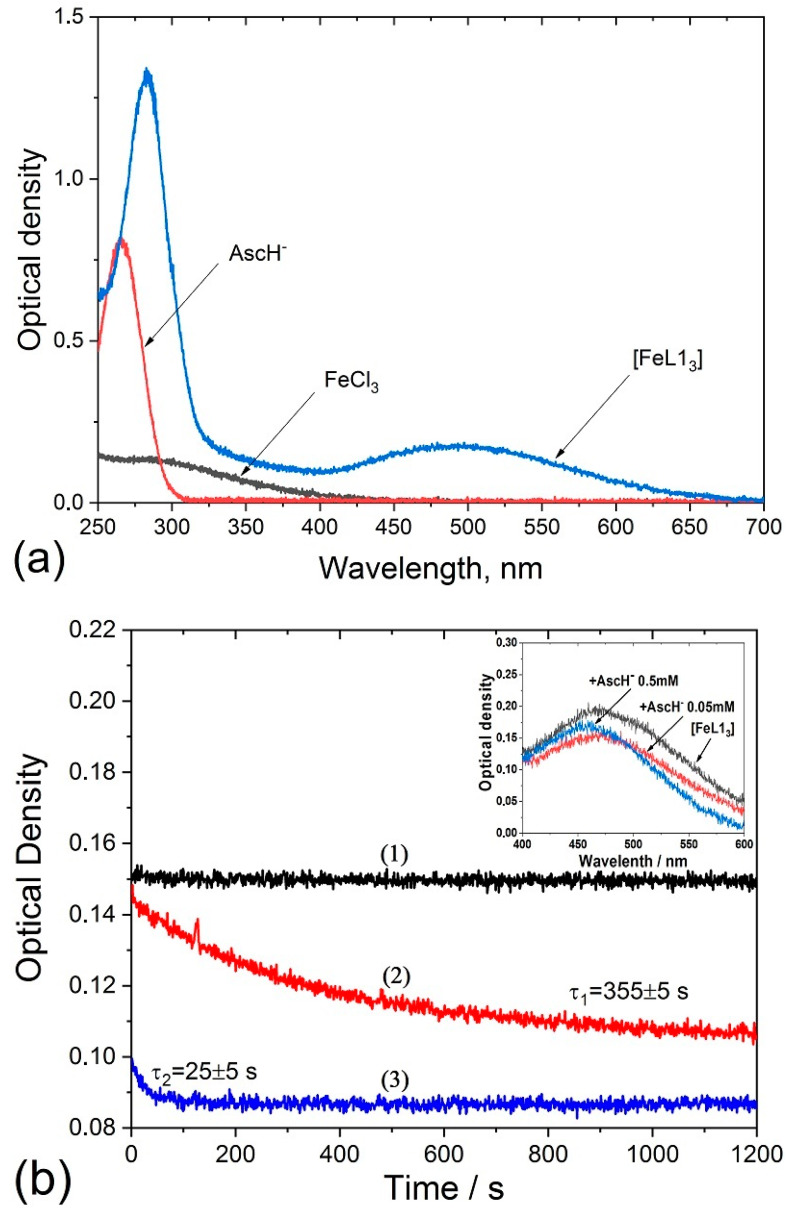

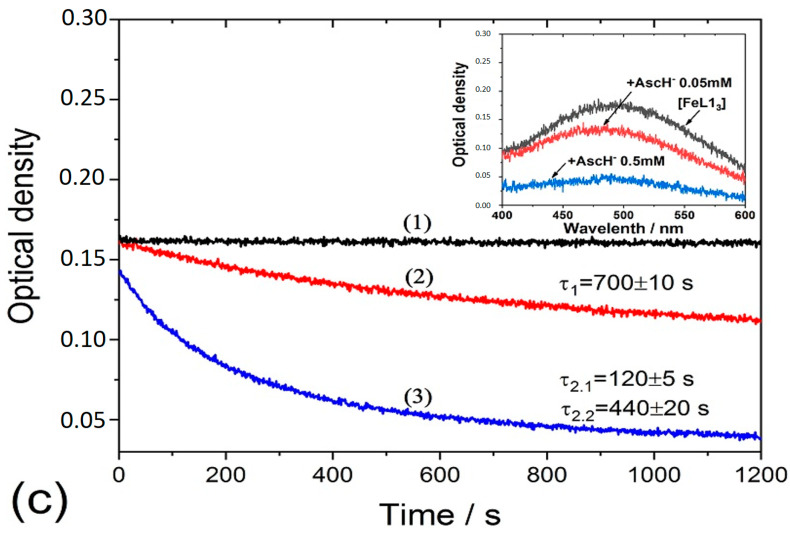
(**a**) Absorption spectra of ascorbic acid (0.05 mM), FeCl_3_ (0.05 mM), and [FeL1_3_] chelate complex (0.05 mM) in a water solution at pH = 4.8; (**b**) time profile of the changes of optical density at 525 nm of FeL1_3_ chelate complex aqueous solution at pH = 7.4 in the absence (1) and presence of ascorbic acid at 0.05 mM (2) and 0.5 mM (3) at room temperature (25 °C); (**c**) similar time profiles measured at pH = 4.2.

As was previously demonstrated in [39], the FeL1_3_ chelate complex has a broad absorption band of around 500 nm in water solution (Figure 2a). After the addition of ascorbic acid, the position of this band was shifted to a lower wavelength (Figure 2b,c). We assume that this change is due to a change in the structure of the chelate complex. To measure the characteristic time of this reorganisation we measured the time profile of changes in optical density of solution at 525 nm after the addition of ascorbic acid. The rate of this process at neutral pH = 7.4 depends on the concentration of ascorbic acid with characteristic times of 350 second (s) at [AscH^−^] = 0.05 mM, and 25 s at [AscH^−^] = 0.5 mM (Figure 2b). However, in a weak acid solution at pH = 4–5 this new complex was unstable, and decayed completely with a characteristic time of several minutes (Figure 2c).

### 2.2. NMR Study of the Interaction of Ascorbic Acid with Iron–Deferiprone Chelate Complex 

**Figure 3:**
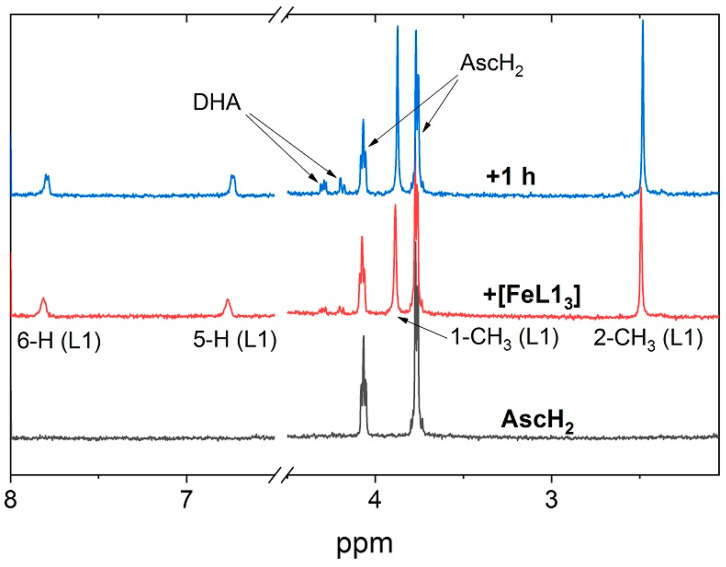
Fragments of the ^1^H NMR spectra of 0.4 mM of ascorbic acid in D_2_O at pH = 4.1 in the absence and in the presence of 0.1 mM of FeL1_3_ complex just after mixing and after one-hour storage at room temperature.

To find out whether the decay of the mixed chelate complex in acidic media results in oxidation of ascorbic acid, we applied the NMR technique. The formation of DHA in the reaction of ascorbic acid with the FeL1_3_ chelate complex in a weak acidic solution was confirmed by ^1^H NMR spectroscopy. Figure 3 shows the appearance of the DHA signal after the addition of FeL1_3_ complex to the ascorbic acid solution, with further increases in the amount of product during the next hour. We can assume that further decomposition of ascorbic acid occurs due to the release of ferrous ions from the L1 complex [39,54] and its oxidation by dissolved oxygen [10]. The concentration of ascorbic acid in this experiment was 0.4 mM, and the concentration of FeL1_3_ was 0.1 mM. 

### 2.3. EPR Spin Trapping Study of the Hydroxyl Radicals Production in the Fenton Reaction and the Effects of Ascorbic Acid and Deferiprone

**Scheme 2:**
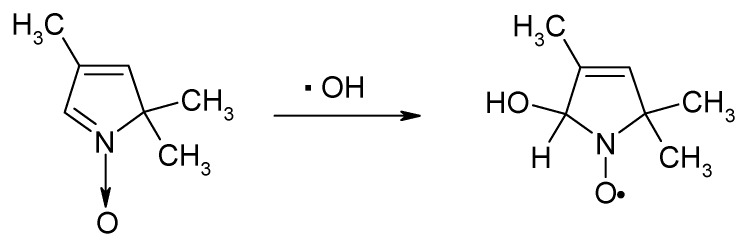
The structures of the TMIO spin trap and spin adduct with OH radical.

The antioxidant/pro-oxidant activities of ascorbic acid were investigated in Fenton type reactions in the absence and presence of L1 using the EPR spin trapping technique. The TMIO spin trap was used for the detection of hydroxyl radicals in these experiments (Scheme 2).

The TMIO spin trap provides more distinguishable individual spectra with the •OH radical than the widely used DMPO spin trap [55,56,57]. In addition, 2H-imidazole 1-oxides do not form superoxide spin adducts in the xanthine/xanthine oxidase system. This is why the use of this spin trap makes the analysis much easier especially where several types of ROS are formed, for example in the Fenton type reaction induced by Cu or Fe ions [58,59,60,61,62,63].

**Figure 4:**
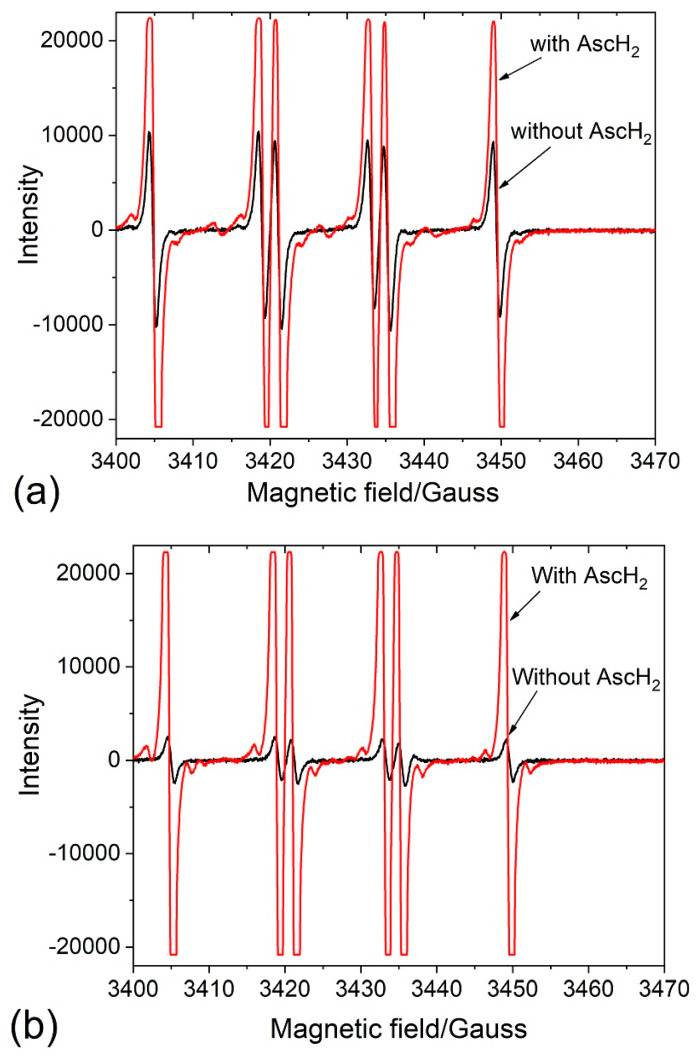
EPR spectra of TMIO–OH spin adduct in the absence (black) and presence (red) of ascorbic acid (**a**) without L1 and (**b**) in the presence of 3 mM L1. Experiments were performed at room temperature in distilled water at pH = 3.3; reaction mixture: 20 mM TMIO; 3 mM FeCl_3_; 12 mM H_2_O_2_; 10 mM ascorbic acid.

The formation of •OH radicals in the systems under study was proved by the detection of the corresponding TMIO–OH spin adduct with HFC constants: a(N) = 14.07 G, a(H) = 16.08 G (Figure 4) [39,40,55,64]. It was found that the signal from •OH spin adducts increased after addition of ascorbic acid to the solution in the absence of L1, as well as in the presence of low concentrations of L1 (L1:Fe = 0 or 1:1, see Figure 4a,b).

**Figure 5:**
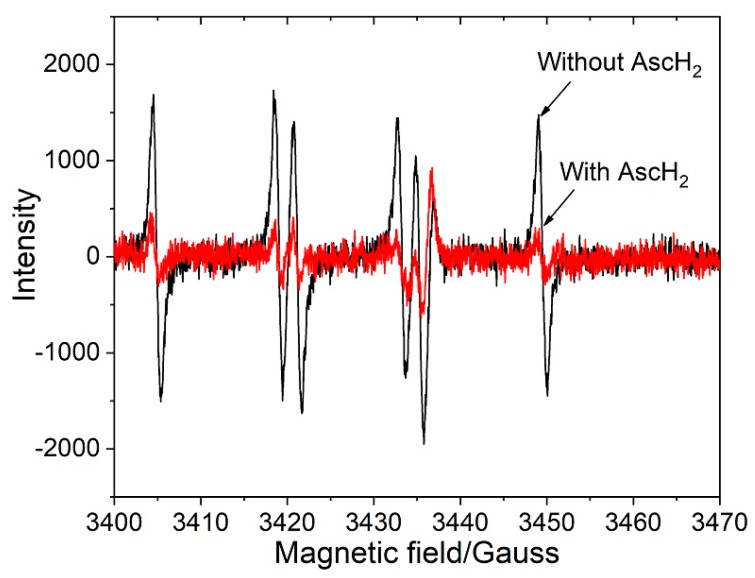
EPR spectra of TMIO–OH spin adduct in the absence (black) and presence (red) of 100 mM ascorbic acid in the presence of 6 mM L1. Reaction mixture: 20 mM TMIO; 3 mM FeCl_3_; 12 mM H_2_O_2_; pH = 3.0.

**Figure 6:**
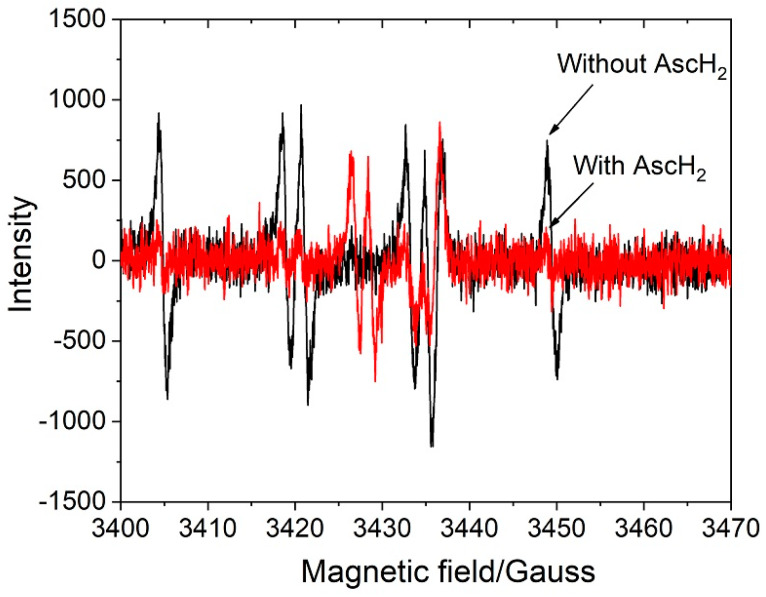
EPR spectra of TMIO–OH spin adduct in the absence (black) and presence (red) of 100 mM ascorbic acid in the presence of 12 mM L1; 20 mM TMIO; 3 mM FeCl_3_; 12 mM H_2_O_2_; pH = 3.0.

However, the situation changed with an increase in the concentration of ascorbic acid from 10 to 100 mM in the reaction mixture. The observed amount of TMIO–OH spin adduct decreased in the presence of ascorbic acid in solution when the concentration ratios of L1:Fe = 2:1 or 4:1 were used (Figure 5 and Figure 6). Although the optical study shows slow decay of the FeL1_3_ complex in the presence of ascorbic acid in a weak acidic solution (see Figure 2c), we can assume the competition in the trapping of the hydroxyl radical by spin trap and ascorbic acid. An additional factor that can affect the intensity of the spin adduct signal is the transformation of the spin adduct to the corresponding hydroxylamine in acidic media. Due to its diamagnetic nature, hydroxylamine is an EPR silent compound. This process becomes important when the rate of hydroxyl radical production decreases due to chelate complexes FeL1_2_Asc or FeL1_3_ formation. 

With an increase of the L1:Fe ratio from 2:1 to 4:1, we observed a further decrease in the signal intensity from the TMIO–OH spin adduct in accordance with our previous studies on the antioxidant activity of L1 [39,40]. Surprisingly, in these conditions, we detected the signal from ascorbyl radicals with EPR parameters: a(H) = 1.8 G (Figure 6) [65].

## 3. Discussion 

The experimental findings of this study have several important biological and physiological implications for understanding the interaction of ascorbic acid with iron and L1. Under certain conditions ascorbic acid causes the reduction of ferric to ferrous iron, resulting in an increase of ROS production, in particular hydroxyl radical production, leading to oxidation to ascorbyl radicals and dehydroascorbic acid. Additionally, ascorbic acid is able to form mixed iron complexes with L1 and possibly with other chelators which may be redox-active or redox-inactive. The significance of these processes is underlined below from the different experiments carried out within this study.

**Scheme 3:**
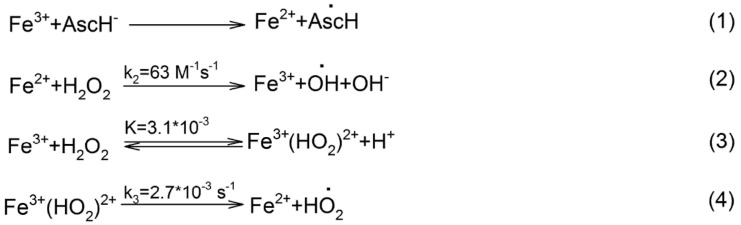
Reduction of Fe^3+^ by ascorbic acid and the Fenton reaction [66].

**Scheme 4:**
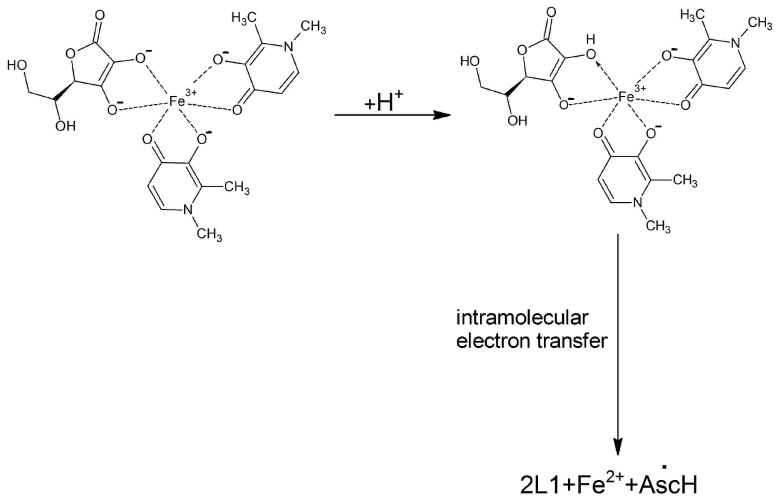
Iron reduction in the mixed chelate complex FeL1_2_Asc.

It is known, that ascorbic acid is oxidised by Fe^3+^ ions through an intermediate chelate complex, forming a stable ascorbyl radical and the redox-active Fe^2+^ ion, which initiates a cascade of redox reactions with the formation of ROS (Scheme 3):

Due to the importance of these processes for living systems, the characteristics of these reactions in various environments attract considerable attention [27,28,29,30,31,32,38,39,40]. Taking into account that at physiological conditions most of the iron is in bound forms, the main question is whether ascorbic acid is able to compete in iron binding with other chelators or transport proteins, following the Fe^2+^ formation. To answer this question, in the present study the behaviour of the FeL1_3_ chelate complex in the presence of ascorbic acid has been studied by UV–Vis optical method at neutral (pH = 7.4) and weak acidic (pH = 4–5) media. FeL1_3_ chelate complex has a broad absorption band of around 500 nm in water solution (Figure 2a). It was found that the addition of ascorbic acid at neutral pH = 7.4 results in shifting the position of this band to a lower wavelength (Figure 2b). We can assume that this change of absorption maximum is due to the substitution of one ligand L1 to ascorbate ion with the formation of a new mixed chelate complex FeL1_2_Asc. The rate of this process is concentration-dependent, with k ≈ 6 M^−1^s^1^. It is important that this mixed chelate complex is redox stable at physiological pH (7.4), no reduction in intensity has been detected in this study. However, in a weak acid solution at pH = 4.0 this new complex was unstable, and decayed completely with a characteristic time of 350 s at [AscH^−^] = 0.05 mM, and ~25 s at [AscH^−^] = 0.5 mM (Figure 2c). We can assume that the reduction of iron in the mixed chelate complex occurs in its protonated form (Scheme 4), similar to that established by Keypour for the Fe(III)–Asc complex [47]. Additionally, the increase of the intensity of L1 proton peaks in the NMR spectrum (Figure 3) is an additional confirmation of the decomposition of the chelate complexes FeL1_2_Asc. The observed effect is due to the decrease of paramagnetic broadening of the L1 NMR signals.

The slow oxidation of ascorbic acid and formation of DHA in the reaction with the FeL1_3_ chelate complex in weak acidic solution was confirmed by ^1^H NMR spectroscopy. Figure 3 shows the appearance of the DHA signal after the addition of FeL1_3_ complex to the ascorbic acid solution, with a further increase in the amount of product during the next hour. We assume that further decomposition of ascorbic acid occurs due to the release of ferrous ions from the L1 complex [39,54] and its oxidation by dissolved oxygen [10]. It should be noted that there are only a few examples of experimentally detected mixed ligand iron complexes of ascorbic acid, but all of them are redox-active, presumably because the chelators involved are weaker than L1 [48,49,50].

The antioxidant/pro-oxidant activity of ascorbic acid was also observed in the present study in the Fenton reaction using the EPR spin trapping technique. The Fenton reactions i.e., the oxidation of organic substrates by Fe^2+^ and hydrogen peroxide has been known for more than a century, and its mechanism was reinvestigated by many others during the recent decades [67,68].

Iron and in particular its ferrous form (Fe^2+^) is the major catalyst of radical reactions in biological systems and the cause of damage in diseases related to oxidative stress. Although no free ferrous iron circulates in blood under normal conditions, the excess “focal” iron was detected in various organs in many diseases [69]. Nontransferrin-bound iron found in plasma is also well documented in iron overloaded diseases like thalassaemia, where transferrin is fully saturated with iron [28]. The presence of labile, nonprotein-bound iron is also considered a source of continuous toxicity, which has been implicated in the pathogenesis of many diseases including ageing, atherosclerosis, neurodegenerative, and kidney diseases, as well as cancer [26].

A number of EPR spin trapping studies have demonstrated the production of reactive oxygen radicals during the oxidation reaction of Fe^2+^ to Fe^3+^ ions with hydrogen peroxide (see Scheme 3) [67,68,70]. In this context, the method of spin trapping allows the transformation of extremely short-lived radicals to long-lived ones, and is successfully used in the study of biologically relevant free radicals, especially ROS, such as hydroxyl radical, peroxyl radical, and singlet oxygen [55,56,57,64,71]. It has been shown that the use of the TMIO spin trap (2,2,4-trimethyl-2H-imidazole-1-oxide) has certain advantages over the widely used DMPO (5,5-dimethylpyrroline-N-oxide) and PBN (α-phenyl N-t-butyl nitrone) spin traps in the trapping of several types of radicals and in the interpretation of molecular mechanisms involved in the reaction [55,56,64].

**Scheme 5:**
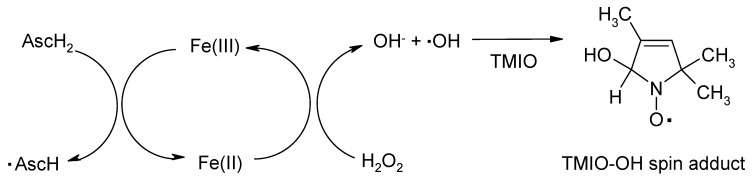
The ascorbic acid enhanced Fenton reaction.

In the present, study the formation of •OH radicals was proven by the detection of the corresponding TMIO–OH spin adduct with hfi (hyperfine interaction) constants: a(N) = 14.07 G, a(H)=16.08 G. It is important to note, that the signal from OH spin adduct increases in the presence of ascorbic acid in the reaction mixture in the absence of L1, as well as at low concentration of L1, as shown in Figure 4a,b. It can be suggested that ascorbic acid turns on the cyclic reaction of •OH radical production (Scheme 5).

Similar effects of a significant increase in the yield of free radicals in cyclic redox reactions have recently been demonstrated in the system containing iron ions and anticancer quinone-chelators [72,73]. The authors also demonstrated the synergetic effect of iron and chelating compounds on cytotoxicity against a number of cancer cell lines. In this context, the pro-oxidant activity of ascorbic acid can be considered as an additional factor in cancer therapy in combination with other anticancer agents and especially with redox-active quinone-chelators [20,72,73,74,75]. Similar cyclic schemes of ROS formation have also been suggested earlier in a carotenoid driven Fenton reaction [62].

In contrast, the amount of TMIO–OH spin adduct decreases in the presence of ascorbic acid in the solution at concentration ratios of L1:Fe = 2:1 and 4:1. So, in these conditions, the antioxidant effect of ascorbic acid has been detected. This experiment confirmed our conclusion drawn from the UV–Vis optical study on the formation of the mixed chelate complex FeL1_2_Asc. This complex is redox inactive in physiological conditions at pH = 7.4, and much less active than Fe(III)–Asc complex in weak acidic media at pH = 3–5. It is known that the stoichiometry of the chelate complex L1 with Fe^3+^ ions is sensitive to pH value. In a slightly acidic medium (pH = 3–5), the redox-active FeL1_2_^+^ complex is also present in solution in equilibrium with FeL1_3_ complex [44]. This observation is in agreement with data published by Devanur with coauthors [63] who observed hydroxyl radical generation (detected using the deoxyribose oxidation assay) in Fenton reaction with participation of the redox-active FeL1_2_^+^ at low concentration of ascorbic acid. 

It is envisaged that different mixed chelate complexes of iron with ascorbic acid and nutrients or drugs with chelating properties such as L1 will be formed in vivo in the gastrointestinal tract which may have implications on iron absorption [76,77,78]. Similarly, in the absence of chelators, the reduction of ferric iron to ferrous iron, which was shown in these studies, once again confirmed the advisability of using ascorbic acid to increase iron absorption, since ferrous iron is better absorbed than ferric iron [76,77,78]. 

It should be noted that ascorbic acid is also able to form a chelate complex with Fe^2+^ ions. The existence of the ferrous ascorbate chelate complex has been established in a number of studies [79,80]. However, this complex cannot be compared in efficiency with other chelating molecules responsible for iron transport, primarily DMT1 (divalent metal transporter 1) [81]. In addition to its well-established reduction of ferric iron to provide a bioavailable supply of ferrous iron to DMT1, ascorbic acid also enhances directly the uptake of ferric iron into Caco-2 cells through the formation of the Fe^3+^–ascorbate complex [82]. Since DMT1 is not involved in the uptake of this complex, further work is required to identify the transporter system (chelating agent) which inhibits Fe^3+^ reduction by ascorbate. 

Considering the possible biological role of ferrous ascorbate complex, the main question is whether this complex is redox-active or not. Some recent studies have confirmed the redox activity of a mixture of ascorbic acid with Fe^2+^ in the Fenton reaction [83,84]. For example, oxidation of benzoic acid by the Fenton reaction occurs more effectively in the presence of ascorbic acid [85]. The initial reactivity of ferrous ascorbate with hydrogen peroxide is the same as that of free ferrous ions. However, when Fe^2+^ is oxidised to Fe^3+^, the reaction rate decreases by one order due to the slow reaction of Fe^3+^ with hydrogen peroxide (see Scheme 3). Furthermore, in the presence of ascorbic acid the starting reaction rate is kept high due to the fast transition of Fe^3+^ to Fe^2+^ [85].

It is worth considering several other biological implications of the interaction of ascorbic acid and iron, such as the prospect of an increase in ROS production and toxicity under conditions of iron loading, especially when using a combination of ascorbic acid and chelating agents. Furthermore, it should also be considered that molecular modification such as oxidation could result in the reduction or complete inactivation of the biological functions of ascorbic acid and its role as a major vitamin.

## 4. Materials and Methods 

### 4.1. Materials 

Ascorbic acid (99%), FeCl_3_ (97%), and H_2_O_2_ (35.5%) were obtained from Sigma-Aldrich, Moscow, Russia. L1 was received from LIPOMED Inc, Arlesheim, Switzerland. The spin trap TMIO (2,2,4-trimethyl-2*H*-imidazole-1-oxide, 98%) used in EPR experiments was obtained from Fluka, Buchs, Switzerland. All compounds were used as received. All experiments were carried out at room temperature. 

### 4.2. Methods

UV–Vis absorption spectra of all compounds and chelate complexes and the kinetic measurements were carried out using a Shimadzu UV-2401-PC spectrophotometer. The samples were prepared in distilled water, and all measurements were made in a 1 cm quartz cuvette. The pH of the solution was adjusted by the addition of HCl or KOH stock solution. 

^1^H NMR spectra were recorded on the Bruker Avance HD III NMR spectrometer (500 MHz ^1^H operating frequency). The samples were prepared in D_2_O (99.9% D, Aldrich) at pH = 4.1.

The EPR spectra of spin adducts were recorded on a Bruker EMX EPR spectrometer. The EPR parameters: microwave bridge (frequency 10.112 GHz, power 20.65 mW) and signal channel (receiver gain 2 × 10^5^, modulation frequency 100 kHz, modulation amplitude 1.00 G). The Fenton reaction was carried out in distilled water (at pH = 3.0–3.5) with 20 mM TMIO, 3 mM FeCl_3_, and at various concentrations of L1, H_2_O_2_, and ascorbic acid. The reaction started with the addition of FeCl_3_ to the reaction mixture. No spin adducts were detected before the addition of FeCl_3_.

## 5. Conclusions

Ascorbic acid is known as a multifaceted compound, and its role in nature and especially in the human body is still the subject of many investigations and debates. On the one hand, ascorbic acid is one of the most important natural substances in the human diet. It performs biological functions as a reducing agent and coenzyme in certain metabolic processes and also serves as an antioxidant. On the other hand, ascorbic acid can act as a chelator of metal ions and also as a source of toxic free radicals in the iron and copper catalysed reactions. This is why the role of ascorbic acid in the iron and copper overload condition is widely discussed and requires further elucidation. Iron is the major catalyst of free radical reactions in biological systems and of free radical damage in the diseases related to oxidative stress. From the results presented in this study, we can conclude that ascorbic acid exhibits predominantly pro-oxidant activity in the presence of iron ions by reducing Fe^3+^ ion to Fe^2+^, thus triggering a cyclic Fenton type reaction, and producing additional amounts of hydroxyl radicals. It has previously been shown in many studies that the chelate complexes of ascorbic acid with ferric iron are redox-active and undergo intramolecular electron transfer with the formation of ascorbyl radicals and Fe^2+^ ions. We propose that the pro-oxidant activity of ascorbic acid in the iron overload condition should be taken into consideration when chelation therapy is applied. In contrast, in the case of cancer therapy, the pro-oxidant activity of ascorbic acid in the presence of iron can be utilised as an additional factor in the combination therapies with other anticancer agents, in particular with redox-active quinone-chelators.

The ability of ascorbic acid to reduce ferric iron to ferrous iron at acidic and neutral pH confirms the suitability of ascorbic acid to increase iron absorption. It was also first established that in the ternary chelate complex of iron, L1, and ascorbic acid (FeL1_2_Asc), ascorbic acid is apparently not able to reduce ferric iron at physiological pH. Therefore, we can assume that ascorbic acid can be used as an antioxidant in cases of L1 chelation therapy. Surprisingly, the formation of a mixed chelate complex occurs even at low concentrations of ascorbate, despite the significantly lower stability constants of the ascorbate complex with iron than the L1 complex. Further investigations are needed for determining the biological effects of ascorbic acid at the molecular level and in particular the effects of mixed iron complexes of iron with ascorbic acid and other chelators.
